# Effect of Oxygen Plasma Treatment on Wheat Emergence and Yield in the Field

**DOI:** 10.3390/plants11192489

**Published:** 2022-09-23

**Authors:** Matej Holc, Miran Mozetič, Rok Zaplotnik, Alenka Vesel, Peter Gselman, Nina Recek

**Affiliations:** 1 Jožef Stefan Institute, Jamova Cesta 39, 1000 Ljubljana, Slovenia; 2 Interkorn Ltd., Gančani 94, 9231 Beltinci, Slovenia

**Keywords:** wheat, plasma, yield, emergence, field, fungicide, eco-layer

## Abstract

This paper investigates the effects of an inductively coupled, radio frequency oxygen plasma on the plant emergence and crop yield of wheat in field growth conditions. Wheat seeds of eight different cultivars were plasma-treated using conditions selected based on preliminary experiments. Additionally, a control sample, as well as seeds treated with fungicide, an eco-layer, or a plasma + eco-layer combination, were planted in parallel. Four cultivars per harvest year were used. Plant emergence (plants/m^2^) and yield (kg/ha) were followed. There was little variation among the control and the various treatments regarding plant emergence. Regarding yield, there were statistically significant differences, but no discernible trend was seen when comparing the individual treatments. In the case of several cultivars, plasma-treated seeds performed as well as the control, but there was a significant increase in yield only in the case of cultivar 88.5 R. In several cases, yield of plants for plasma-treated seeds was also lower than the control. Our results demonstrate that the response of wheat yield to plasma treatment, as well as to other seed treatments, differs depending on the wheat cultivar.

## 1. Introduction

Crop yield is one of the most important agricultural parameters, as it directly describes the amount of crop produced per area of land. An increase in yield has historically allowed for higher agricultural productivity and, thereby, important socio-economic developments. It is the focus of many agricultural improvement strategies, including the use of fertilizers, pesticides, and genetic modification of organisms [1].

In the science of plasma agriculture, the technology of non-equilibrium gaseous plasma is applied in agricultural contexts. When crop seeds are directly treated by plasma, the aim is typically to achieve improvement of germination and early growth, decontamination, increased resistance to environmental stress, and, ultimately, yield improvement in field growth experiments [2]. Plasma is an environmentally benign technology, and its use in agriculture is considered to supplant or supplement the use of harmful chemical methods of production increase.

After treatment of agricultural seeds with plasma devices, it is common to evaluate plasma effects on the germination and growth parameters of the seeds and resulting seedlings. However, the effects on the crop yield itself are not considered nearly as often. Wheat appears to be the most well-researched crop in the sense of plasma treatment of seeds for agricultural purposes [3], but out of over 50 literature contributions that treat wheat seeds with gaseous plasma, only 10 consider the plasma treatment effect on the actual yield parameters. An overview of the plasma effects on wheat yield currently available in the literature is shown in Table 1 below.

The earliest available study of wheat yield to also employ plasma was by Marinković and Borcean [14]. However, the paper mainly focuses on the effects of extremely low-frequency electromagnetic fields, and appears to have used plasma mainly for disinfection purposes. No details of the used plasma system are provided, and the plasma exposure alone did not affect wheat yield.

Out of the papers collected in Table 1, initial experiments were generally performed in a laboratory setting, where any changes to germination, seedling growth, surface properties, and biochemistry were also measured. Yield, along with plant growth parameters, was generally determined in real field conditions. One exception is the contribution of Saberi et al., where yield was determined in greenhouse experiments under haze stress conditions [11]. The field experiments were performed on a plot of different sizes, from a few m^2^ [5,9,12] to as much as 6 ha [7] per treatment condition.

In the available literature, the yield of wheat after plasma treatment of seeds was recorded in several ways. Most of the available literature considers grain yield, typically expressed as g/m^2^ or t/ha [5,6,7,8,9,10]. Additionally, 1000-grain mass was also commonly reported [4,5,6,8,9,10,12,13]. Some authors further emphasized yield components such as the number of grains per spike [5,6,8,9,13], number of spikelets per spike, length of spike [8,9], or spikes per plot [6].

Several of the available papers used low pressure, capacitively coupled (CC) radio frequency (RF) plasma systems to treat wheat seeds. Jiang et al. using helium as the process gas, achieved a nearly 6% increase in grain yield, along with improvements to growth parameters in the field, such as plant height, fresh weight, stem diameter, and leaf area and thickness. The laboratory germination rate also increased [7]. Hui et al. used an air/He mixture, and while yield components such as 1000-grain mass, grains per spike, and spikes per plot did not significantly increase, the theoretical and actual yield still improved up to around 8% in the first generation. The germination rate, as well as seedling and plant growth parameters, also improved [6]. Zhang et al. used both air and air/He and improved both the 1000-grain mass and the number of grains per spike. In addition, they noted increases in the germination rate, shoot and root length of seedling, and plant growth parameters, as well as changes in enzyme activity [13]. In both their works, Saberi et al. used air as the process gas. In the case of field growth, the treatment increased both grain and biological yield by over 30 %, along with 1000-grain mass [10]. In the greenhouse experiment under haze stress conditions, the treatment improved grain and spike yield of winter wheat, expressed in grams per plant, for 58 and 74%, respectively [11]. Both treatments also induced changes to several biochemical parameters. Another group working with winter wheat, Filatova et al. also used CC RF air plasma but only achieved modest increases, around 1–2%, to yield and 1000-grain mass. The treatment also left the germination rate unaffected, increased sprout and root length, and decreased both the degree of fungal infection and disease severity in the field [4].

The remaining contributions, performed using discharges at higher pressures, were carried out by the same research group. In three of the papers, the authors sustained their discharges at a medium pressure of about 10 Torr. Hasan et al. used a dielectric barrier discharge (DBD) sustained in air and achieved increases in grain yield, 1000-grain mass, and the number of grains per spike. The group also detected surface etching and cracks by scanning electron microscopy (SEM), increases in the germination rate as well as seedling and plant growth, and changes in biochemistry and enzyme activity [5]. Roy et al. used a glow discharge in air or air/O_2_, and found a 20% increase in yield, along with significant improvements to 1000-grain mass, length of a spike, and the number of spikelets and grains per spike. They observed modifications to the surface using SEM, and noted increased water uptake of seeds. The germination rate, plant length, and growth parameters also increased [8]. Sohan et al. also used a glow discharge, but in the air or Ar/O_2_, and only recorded an improved 1000-grain mass. In addition, they noted etching through SEM, increased germination rate, shoot and root length, and growth parameters, as well as changes to biochemistry and enzyme activity [12]. The only contribution to the use of atmospheric pressure plasma was a second paper by Roy et al., who used a gliding arc discharge in air/O_2_/H_2_O. The treatment increased yield by 20 % and further increased 1000-grain mass, length of a spike, and the number of spikelets and grains per spike. Water uptake, germination rate, plant length, and growth parameters also increased [9].

Notably, regardless of the plasma system used, several of the available papers found an increase in either photosynthetic activity [10,11] or chlorophyll concentration [5,7,8,9].

In our own experiments, we have worked with eight cultivars of wheat: Alixan, Genius, Nexera 88, and Sofru in the first year, and Bologna, Izalco, Amicus, and 88.5 R in the second year. Regarding yield parameters, we have recorded emergence (no. of plants per m^2^) and yield (kg/ha). We have compared the field growth of wheat from untreated, control seeds with that from plasma-treated seeds, as well as seeds with other treatments. The seeds were treated in an industrial-size plasma reactor which allowed the treatment of 1 kg of seeds per batch.

## 2. Results and Discussion

### 2.1. Emergence of Plants in the Field

**Figure 1:**
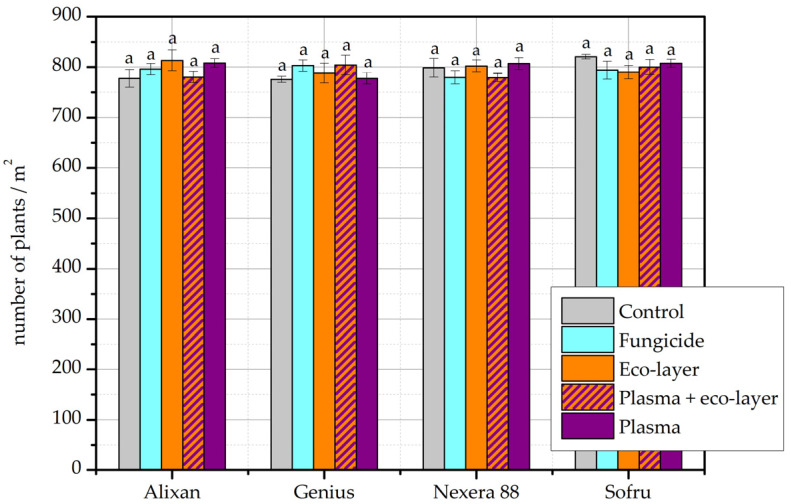
The number of emerged plants per m^2^ according to individual treatments and cultivars in the year 2019/20. The error bars represent standard error. Different lowercase letters above data points represent statistically significant differences (*p* < 0.05; post hoc Tukey’s range test) between treatments.

The average number of emerged plants per m^2^ for cultivars Alixan, Genius, Nexera 88 and Sofru in the year 2019/20 is shown in Figure 1. From the obtained results, we can see that there are no major differences between the treatments or the different cultivars, and no similar trend among the different treatments is observed. On average, the number of emerged plants varies between 780 and 810. Cultivars Alixan and Genius performed slightly better with all treatments compared to the untreated group, however, there is no significant difference in the number of emerging plants between any of the treated groups and/or control. Interestingly, cultivar Sofru shows the highest number of emerging seeds in the control group, where no plasma, no eco-layer or pesticides were applied to seeds. Based on the results, we can conclude that the wheat treated with the eco-layer performs as well as the wheat treated with a fungicide. The same applies to wheat that was treated with plasma. None of the differences between individual treatments of the same wheat cultivar were statistically significant.

**Figure 2:**
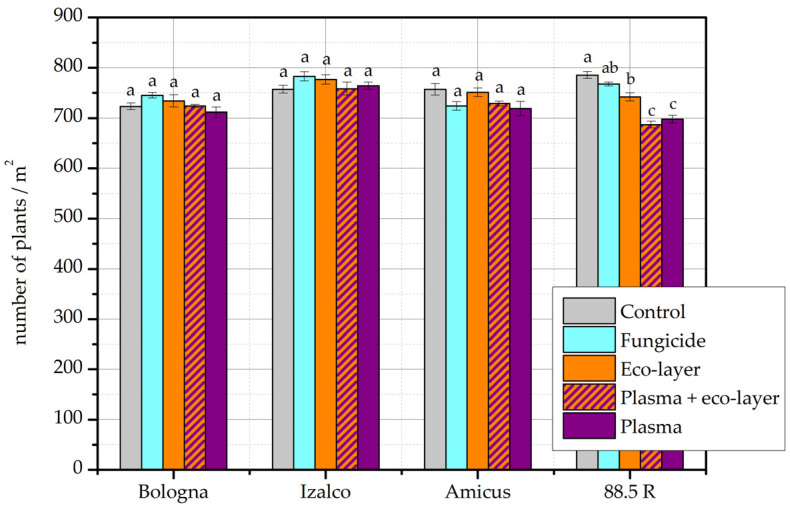
The number of emerged plants per m^2^ according to individual treatments and cultivars in the year 2020/21. The error bars represent standard error. Different lowercase letters above data points represent statistically significant differences (*p* < 0.05; post hoc Tukey’s range test) between treatments.

Figure 2 shows the number of emerged plants in the year 2020/21. On average, the number varies between 720 and 780. For the cultivars Bologna, Izalco and Amicus, there were minor differences between treatments, but none were statistically significant. For the cultivar 88.5 R, however, the control and fungicide treatments were comparable, while the control sample emergence was significantly higher than the remaining three treatments. Additionally, the fungicide and eco-layer treatments were comparable, but had higher emergence than the plasma and plasma + eco-layer treatments, which were also comparable.

From the obtained results, we see that the emergence of plants in the field was not significantly affected by any of the applied treatments. For the cultivars planted in the year 2019/20 in particular, the emergence was very comparable across treatments and cultivars (Figure 1). For the cultivars planted in the year 2020/21, the differences were somewhat more pronounced (Figure 2). According to results of two-way ANOVA, only the number of plants for the cultivar Izalco differed significantly from all other cultivars, which were comparable.

In the available literature where authors investigated wheat yield after plasma treatment of seeds, the emergence of plants in the field was not followed. However, some authors followed the laboratory germination rate of seeds, which correlates well with field emergence, at least under favorable soil conditions [15]. In several publications, the same plasma treatment conditions that resulted in increased yield have also been shown to increase the germination rate [5,7,8,12]. However, germination may also remain unchanged, or even decrease, depending on plasma treatment specifics [4]. When the wheat seed germination rate is already high, as in the case of certain commercial cultivars, additional treatments may not result in an additional increase [3], and as such, to increased emergence in the field. However, as seen in Figure 2, in the case of cultivar 88.5 R, wheat plant emergence is significantly higher for seeds of the control group compared to any of the applied treatments, including plasma. This may relate to the specific properties of this particular cultivar.

### 2.2. Yield of Wheat Seeds after Harvest

**Figure 3:**
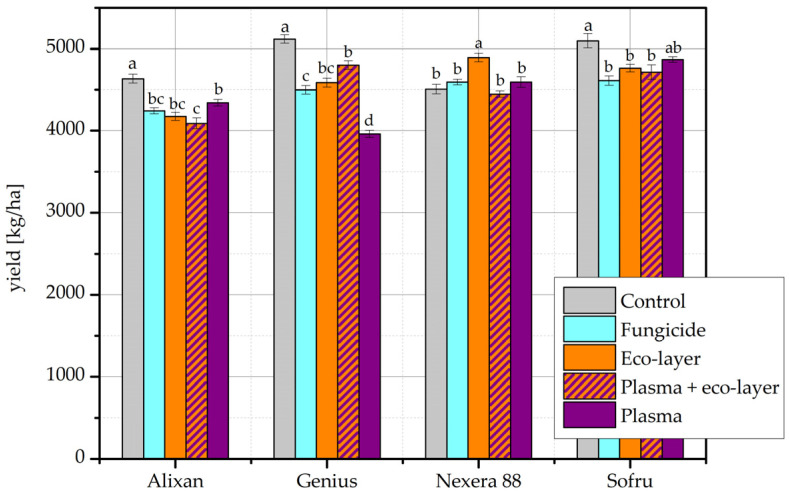
Yield results (kg/ha) of the field experiment according to individual treatments and cultivars of wheat in the year 2019/20. The error bars represent standard error. Different lowercase letters above data points represent statistically significant differences (*p* < 0.05; post hoc Tukey’s range test) between treatments.

The results of the harvest in the year 2019/20, specifically mid-August 2020, are shown in Figure 3. From the obtained results, it is clear that there are no major deviations between the treatments nor between the cultivars. With the exception of the cultivar Nexera 88, the highest yield was obtained from untreated control seeds. For the cultivar Alixan, the control yield was significantly higher than all the remaining yields, while the plasma yield was additionally significantly higher than the plasma + eco-layer yield. For the cultivar Genius, the control yield was also significantly higher than all the remaining yields. Additionally, the fungicide, eco-layer, and eco-layer + plasma yields were all significantly higher than the plasma yield alone, and the eco-layer + plasma yield was significantly higher than the fungicide yield. In the case of Nexera 88, the eco-layer treatment achieved the highest yield, which was significantly higher than all of the remaining yields. Finally, for the cultivar Sofru, the control yield was significantly higher than the fungicide, eco-layer, and eco-layer + plasma yields, but comparable to the plasma yield. The seeds treated with O_2_ plasma produced an average of all four cultivars of 4500 kg/ha of grain, where the highest yield was measured in the cultivar Sofru, followed by Nexera 88, Genius, and Alixan.

**Figure 4:**
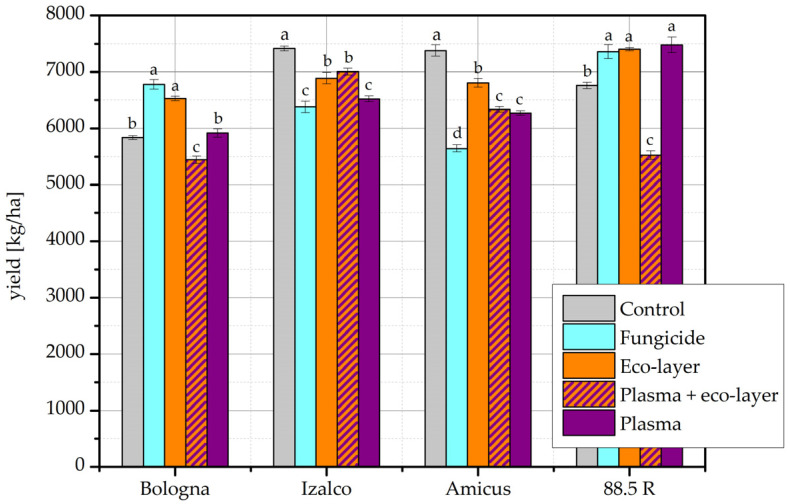
Yield results (kg/ha) of the field experiment according to individual treatments and cultivars of wheat in the year 2020/21. The error bars represent standard error. Different lowercase letters above data points represent statistically significant differences (*p* < 0.05; post hoc Tukey’s range test) between treatments.

Figure 4 represents the results of the wheat harvest in the year 2020/21. For the cultivar Bologna, yield from plasma-treated seeds was comparable to yield from control seeds, and fungicide yield was comparable to eco-layer yield; the remaining differences were statistically significant. For the cultivar Izalco, the eco-layer and eco-layer + plasma yields were comparable, as were the plasma and fungicide yields; the remaining differences were statistically significant. For the cultivar Amicus, all values differed significantly from one another, with the exception of the yields from plasma- and plasma + eco-layer-treated seeds, which were comparable. For the cultivar 88.5 R, the plasma, eco-layer, and fungicide yields were comparable, but significantly higher than the control yield, which was in turn significantly higher than the plasma + eco-layer yield. The highest yields were observed in cultivar Izalco, with untreated seeds, and cultivar 88.5 R, with plasma-treated seeds as well as the fungicide and eco-layer treatments.

As seen above, the yield results are not uniform, and appear to depend on the specific wheat cultivar. To the best of our knowledge, none of the available literature regarding plasma effects on wheat yield uses more than one wheat cultivar, and none uses any of the cultivars used in our experiments. Wheat cultivars differ from one another in numerous properties, including yield potential, but also seed characteristics such as size, weight, and composition [16]. Thus, wheat cultivars respond to treatments, including plasma, differently. It has been previously shown that two wheat cultivars can respond differently to the same plasma treatment regarding etching and nanostructuring of the seed surface, as well as the germination rate and seedling growth parameters [17]. Here, we demonstrate that the response of wheat yield to plasma treatment, as well as to other seed treatments, may also differ depending on the wheat cultivar. This is confirmed by the results of two-way ANOVA, in which significant differences between the cultivar results were obtained. In the year 2019/20, results for all cultivar pairs differed significantly, with the exception of Genius and Nexera 88, which were comparable. Similarly, in the year 2020/21, results for all cultivar pairs differed significantly, with the exception of Izalco and 88.5 R, which were comparable.

As seen in Table 1, when wheat yield in the available literature was measured in terms of mass per field area, plasma treatment typically increased the yield compared to the control [5,6,7,8,9,10,11]. Such an increase was not achieved in our own experiments. In the year 2019/20, unfavorable weather conditions contributed to low yields; harvesting of wheat was late due to heavy summer rainfall, and the final harvest yields were lower than average. Control yields were significantly the highest for three out of the four cultivars planted that year. In the year 2020/21, more notable improvements were seen with certain treatments for the cultivars Bologna and 88.5 R. The most useful improvement of yield by plasma treatment alone appears to be the result of the cultivar 88.5 R (Figure 4), where plasma treatment resulted in a yield significantly higher than the control, but comparable to the treatments of eco-layer and fungicide.

None of the experiments in the available literature used inductively coupled RF plasma, though several did use capacitively coupled RF plasma [4,6,7,10,11,13]. It is established that different plasma systems or treatment conditions may have different effects on the properties of the treated seeds, including the germination rate and seedling growth parameters [18]. It is therefore plausible that the specifics of the plasma treatment used in these experiments affect wheat seeds in a way that disallows yield improvement.

Considering all available results, the individual treatments do not affect the plant emergence and yield of a specific cultivar in the same way. For example, for the cultivar 88.5 R, the yield was the highest when seeds were treated with plasma alone, but emergence for the same cultivar and treatment was significantly lower than the control emergence.

### 2.3. Correlation between Plant Emergence and Yield

**Figure 5:**
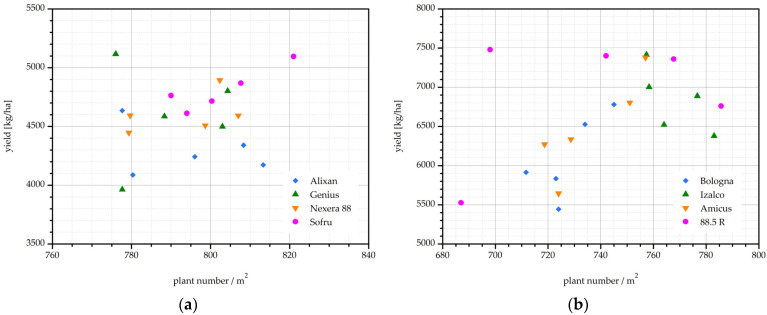
Correlation between plant emergence / m^2^ and yield (kg/ha) of the field experiments in the years (**a**) 2019/20 and (**b**) 2020/21.

The correlation between plant emergence and yield is illustrated in Figure 5 for each harvest year separately. For some of the individual cultivars, such as Sofru in Figure 5a or Amicus and Bologna in Figure 5b, there appears to be a reasonably linear correlation between the two yield parameters. These are also the cultivars with the highest correlation coefficients, namely r = 0.892, 0.871, and 0.773, respectively. For the remainder of the cultivars, and by extension for the experiments in general, the results are quite scattered, and the correlation coefficients are lower. For all the results combined, the correlation coefficient is quite low, r = −0.582.

## 3. Materials and Methods

### 3.1. Seed Material

Seeds of wheat cultivars Alixan, Genius, Nexera 88, Sofru, Bologna, Izalco, Amicus, and 88.5 R were obtained from Interkorn Ltd. (Gančani, Slovenia). Immediately after storage, plasma treatment of seeds was performed. Some seeds were further subjected to eco coating, as described below. All of the seeds, with and without eco-layer applied, were sown two days after treatment. Each cultivar was sown in 20 plots in 4 replications, meaning 80 plots in total. Wheat seeds were sown and harvested independently by Žipo Lenart Ltd. (Lenart, Slovenia) and the Agricultural Institute of Slovenia (Ljubljana, Slovenia) for two consecutive years, 2019/20 and 2020/21.

### 3.2. Plasma Reactor

**Figure 6:**
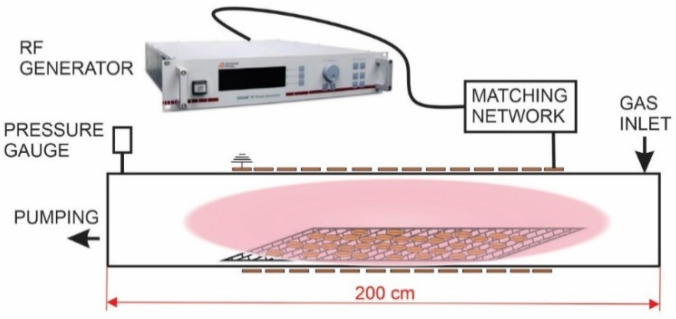
Schematic representation of industrial plasma reactor used to treat wheat seeds.

The experimental setup used in this study is presented in Figure 6. A 20 cm in diameter and 2 m long glass discharge tube was pumped with a two-stage rotary vacuum pump (Leybold Trivac D65B) which has a nominal pumping speed of 65 m^3^/h. Gas was leaked through mass flow controllers (Advanced energy Aera FC7700) on the opposite side of the pumping. Oxygen with 99.999% purity was used. The pressure was measured with an absolute capacitance pressure gauge (MKS Baratron 722B).

Inductively coupled plasma was generated inside a 14-turn excitation coil connected to a 5 kW, 13.56 MHz RF generator (Advanced energy Cesar 1350) through an in-house made L-type matching network. In all the experiments, the forward RF power was set to 2000 W, whereas the reflected power was around 750 W.

In order to make the manipulation of wheat grains easier, a perforated aluminum holder was used.

### 3.3. Plasma Treatment

During each treatment, 1 kg of wheat seeds was treated simultaneously. The dry seeds were placed into a perforated aluminum tray, which was placed in the middle of the copper coil, as shown in Figure 6. The E-mode plasma was fairly uniformly distributed along the discharge tube. Seeds were directly exposed to the glowing oxygen plasma. The discharges were ignited at a pressure of 40 Pa. The output power at the generator was 2000 W, and the reflected power was 750 W for the E-mode, so the real power absorbed by the plasma was 1250 W. Treatment time was fixed to 120 s, whereas longer treatment times caused visible damage to the seeds during preliminary experiments and were thus not considered for inclusion.

### 3.4. Eco Coating Application

Eco coating was prepared prior to treatment in one of the following ways. In the first case, it was made by diluting the suspension of *Bacillus subtilis* with water in the appropriate ratio by applying 0.6 g of the bacterium *Bacillus subtilis* (0.5% suspension) and 0.1 mL of the algae *Ascophyllum nodosum* per 100 g of wheat seeds. In the second case, the eco-layer was the commercially available Sonata (Bayer, Leverkusen, Germany)—*Bacillus pumilus* + glycerol.

Eco coating was applied to untreated seeds and to plasma-treated seeds three days after plasma treatment due to the logistic between the Institute Jožef Stefan, where the plasma treatment of seeds was performed, and Interkorn Ltd., where seeds were further subjected to eco coating. Seeds were left to dry, then packed in paper bags and sown two days after applying the eco coating.

### 3.5. Experimental Design

Wheat seeds of each of the 8 cultivars were treated for a total of 20 kg per cultivar. Each cultivar was subjected to 4 treatments plus an untreated group, a total of 5 kg of seeds per group. The first group was untreated seeds (no plasma treatment, no fungicide, no eco-layer). The second group was treated only with the commercial fungicide Redigo Pro (Bayer, Leverkusen, Germany), the third group with an eco-layer, the fourth group was pre-treated with plasma and then with an eco-layer, and the fifth group was only plasma-treated.

In the year 2019/20, based on our previous studies and studies from other groups, we designed a first field experiment with cultivars of wheat in the field of Žipo Lenart Ltd., starting in autumn 2019. The experiment was based on five different treatments and four cultivars, namely Alixan, Sofru, Genius, and Nexera 88. Each cultivar/treatment combination had four replications, meaning a total of 80 plots in the experiment. Treated seeds were sown in the soil in October 2019, and the number of emerged plants was counted in the field separately in each plot in April–May next year. In mid-August 2020, with the help of Žipo Lenart Ltd. and the Agricultural Institute of Slovenia, wheat in all 80 plots was harvested.

In the following year, 2020/21, due to unpromising results of plant emergence and yield of all four cultivars of wheat in the year 2019/20, four different cultivars of wheat seeds were chosen: Bologna, Izalco, Amicus and 88.5 R. The main purpose was to determine if plasma has different effects on different cultivars, as well as to cover a wide range of wheat cultivars and determine the effect of plasma on them. A different eco-layer (*Bacillus pumilus* + glycerol) was applied as well, mainly due to its better performance in laboratory conditions, which was to be confirmed in the field experiment. The field experiment was planned in the same manner as the previous year: five different treatments and four cultivars, each with four replications, adding up to 80 plots. We monitored the yield of emerged wheat in mid of August 2021, during the harvesting season.

### 3.6. Emergence of Plants in the Field

The emergence of seeds, in no. of plants per m^2^, was monitored and counted in real-life conditions on the field. Eight different cultivars of seeds were planted in 80 plots, 20 plots for each cultivar of wheat seeds. The size of each plot was 6 m × 1.3 m. One kg of each group of treated or untreated seeds was planted on every plot in 4 replications. None of the seed groups was watered before, during, or after plant emergence, meaning all the groups were growing in the same conditions, depending only on the weather. The emergence of plants was counted on 3 of the plots for each group of seeds. The average number of emerged plants was calculated from the number of counted plants on three plots of the same cultivar.

### 3.7. Yield of Wheat Seeds after Harvest

The yield of all eight cultivars of wheat seeds was separately collected after harvest, and the average yield of each wheat cultivar on all four plots was calculated in kg/ha. Since each cultivar was subjected to 5 different treatments, every treatment of each cultivar (in 4 replications) was collected separately, and the yield was determined.

### 3.8. Statistical Analysis

Data from the plant emergence and yield measurements were statistically analyzed using the JASP 0.9.2. open-source software (University of Amsterdam, Amsterdam, The Netherlands). Group means were calculated and compared using one-way and two-way ANOVA followed by the post hoc Tukey’s range test. Differences in the means were considered statistically significant at (*p* < 0.05). Where error bars are shown in charts, they represent standard error. Correlation coefficients were calculated using the Data Analysis ToolPak available in Microsoft Excel (Microsoft Corporation, Redmond, WA, USA).

## 4. Conclusions

In this work, we report the results of a two-year field experiment. Each year, four different cultivars of wheat were used in the experiment, and four different treatments were applied in addition to an untreated control group, including a plasma treatment, as well as a plasma treatment with additional application of an eco-layer. We followed the different treatment conditions in the field with regard to plant emergence and yield.

With one exception, the results of plant emergence showed no significant difference in the number of emerging plants between the treated groups and/or control; wheat treated with an eco-layer performed as well as wheat treated with a fungicide or treated with plasma. In the case of cultivar 88.5 R, all treatments performed significantly lower than the control. With yield, measured in kg/ha, we have shown that results differ based on the wheat cultivar. No obvious improvement in plant emergence or yield was observed with the selected cultivars and treatment conditions. Plasma treatment itself, which has previously been shown to be beneficial to wheat yield, was here found to be comparable to treatments such as fungicide or eco-layer application. Again, this response was dependent on the specific wheat cultivar.

## Figures and Tables

**Table 1:** An overview of publications regarding the effects of plasma treatment on wheat yield. CC: capacitively coupled, RF: radio frequency, DBD: dielectric barrier discharge, GR: germination rate, WU: water uptake, S: sprout, R: root, SEM: scanning electron microscopy, ↑: increase, ↓: decrease, unch.: unchanged, n/a: not available.

Author	Year	Plasma	Gas	*p* [Pa]	Yield	↑ Yield [%]	Other Effects	Plot	Plot per Condition	Stress
Filatova [4]	2020	CC RF	air	200	↑ yield, ↑ 1000-grain weight	2.3	unch./↓ GR, ↑ S/R, ↓ % fungal inf., ↓ disease severity (field)	field	25 m^2^	fungi
Hasan [5]	2022	DBD	air	1333	↑ yield (g/m^2^), ↑ 1000-grain weight, ↑ grains/spike	27.06	etching, cracks (SEM), ↑ GR, ↑ S/R, ↑ growth, ↑ chlorophyll, biochem. changes, enzyme activity	field	4 m^2^	n/a
Hui [6]	2020	CC RF	air/He	130–160	↑ yield (theoretical, actual) (kg/plot), unch, 1000-grain weight, unch. grains/spike, unch. spikes/plot	8.12	↑ GR (1st gen.), ↑ S, unch. R, ↑ growth	field	660 m^2^	n/a
Jiang [7]	2014	CC RF	He	150	↑ yield (t/ha)	5.9	↑ GR, ↑ R, ↑ growth, ↑ chlorophyll	field	6 ha	n/a
Roy [8]	2017	glow	air, air/O_2_	1333	↑ yield (t/ha), ↑ length of spike, ↑ spikelets/spike, ↑ grains/spike, ↑ 1000-grain weight	~20	altered surface (SEM), ↑ WU, ↑ GR, ↑ plant length, ↑ growth, ↑ chlorophyll	field	n/a	n/a
Roy [9]	2018	gliding arc	air/O_2_/H_2_O	atm	↑ yield (g/m^2^), ↑ length of spike, ↑ spikelets/spike, ↑ grains/spike, ↑ 1000-grain weight	~20	↑ WU, ↑ GR, ↑/unch. plant length, ↑ growth, ↑ chlorophyll	field	5 m^2^	n/a
Saberi [10]	2018	CC RF	air	10	↑ yield (grain, biological) (g/m^2^), ↑ 1000-grain weight	31.62	↑ photosynthesis, biochem. changes	field	20 rows, 6 m	n/a
Saberi [11]	2019	CC RF	air	low	↑ yield (grain, spike) (g/plant)	58	↑ photosynthesis, biochem. changes	greenhouse	3 pots, 3 seedlings /pot	haze
Sohan [12]	2021	glow	air, Ar/O_2_	1333	↑ 1000-grain weight	n/a	etching (SEM), ↑ GR, ↑ S/R, ↑ growth, ↑ chlorophyll, biochem. changes, enzyme activity	field	4 m^2^	n/a
Zhang [13]	2018	CC RF	air, air/He	30–200	↑ 1000-grain weight, ↑ grains/spike	n/a	↑ GR, ↑ S/R, ↑ growth parameters, enzyme activity	field	n/a	drought

## Data Availability

Not applicable.
